# A Novel Fatty Acyl Desaturase from the Pheromone Glands of *Ctenopseustis obliquana* and *C. herana* with Specific *Z*5-Desaturase Activity on Myristic Acid

**DOI:** 10.1007/s10886-013-0373-1

**Published:** 2014-01-11

**Authors:** Åsa K. Hagström, Jérôme Albre, Leah K. Tooman, Amali H. Thirmawithana, Jacob Corcoran, Christer Löfstedt, Richard D. Newcomb

**Affiliations:** 1 Pheromone Group, Department of Biology, Lund University, Sölvegatan 37, 223 62 Lund, Sweden; 2 The New Zealand Institute for Plant & Food Research Limited, Auckland, New Zealand; 3 The Allan Wilson Centre for Molecular Ecology and Evolution, University of Auckland, Auckland, New Zealand; 4 The School of Biological Sciences, University of Auckland, Auckland, New Zealand

**Keywords:** Pheromone biosynthesis, *Ctenopseustis*, Fatty acyl desaturase, Tortricidae, Tetradecanoic acid

## Abstract

Sexual communication in the Lepidoptera typically involves a female-produced sex pheromone that attracts males of the same species. The most common type of moth sex pheromone comprises individual or blends of fatty acyl derivatives that are synthesized by a specific enzymatic pathway in the female’s pheromone gland, often including a desaturation step. This reaction is catalyzed by fatty acyl desaturases that introduce double bonds at specific locations in the fatty acid precursor backbone. The two tortricid moths, *Ctenopseustis obliquana* and *C. herana* (brown-headed leafrollers), which are endemic in New Zealand, both use (*Z)*-5-tetradecenyl acetate as part of their sex pheromone. In *C. herana,* (*Z*)-5-tetradecenyl acetate is the sole component of the pheromone. Labeling experiments have revealed that this compound is produced via an unusual Δ5-desaturation of myristic acid. Previously six desaturases were identified from the pheromone glands of *Ctenopseustis* and its sibling genus *Planotortrix*, with one differentially regulated to produce the distinct blends used by individual species. However, none were able to conduct the Δ5-desaturation observed in *C. herana*, and presumably *C. obliquana*. We have now identified an additional desaturase gene, *desat7*, expressed in the pheromone glands of both *Ctenopseustis* species, which is not closely related to any previously described moth pheromone desaturase. The encoded enzyme displays Δ5-desaturase activity on myristic acid when heterologously expressed in yeast, but is not able to desaturate any other fatty acid (C8–C16). We conclude that *desat7* represents a new group of desaturases that has evolved a role in the biosynthesis of sex pheromones in moths.

**Electronic supplementary material:**

The online version of this article (doi:10.1007/s10886-013-0373-1) contains supplementary material, which is available to authorized users.

## Introduction

The ability of potential mates to locate one another is essential in sexually-reproducing species. Most Lepidoptera use a sophisticated chemical communication system for localization, where female moths emit volatile sex pheromones that attract conspecific males. Generally moths utilize multi-component pheromone blends consisting of fatty acid derivatives, usually alcohols, aldehydes, or acetates (Blomquist et al. 2005; Tillman et al. 1999), which are synthesized in a specialized abdominal pheromone gland in female moths. Fatty acids such as stearic and palmitic acid are processed by various enzymes, the first of which are generally fatty acyl desaturases that introduce one or more double bonds at specific locations in the carbon chain (Blomquist et al. 2005; Jurenka 2004). The fatty acyl desaturases that are used in pheromone biosynthesis generally are thought to have evolved from a conserved metabolic Δ9-desaturase that exists in all eukaryotes (Liu et al. 1999). Moths have an additional Δ9-desaturase that prefers oleic acid over palmitic acid compared to the metabolic desaturases, which prefer palmitic over oleic acid (Park et al. 2008; Rodriguez et al. 2004). The largest desaturase lineage in moths comprises the Δ11-desaturases that contain many enzymes involved in pheromone biosynthesis. The origin of this lineage, presumably from Δ9-desaturases, predates the radiation of the ditrysian moths (Liénard et al. 2008), and its expansion has resulted in the evolution of many new desaturases, some with altered activities, that are used to produce sex pheromone components in moths (for examples see Ding et al. 2011; Liénard et al. 2010). Chain shortening of fatty acyl moieties before or after desaturation is postulated to be catalyzed by one or several β-oxidases. The acids then are reduced to alcohols by fatty acyl reductases (Moto et al. 2004), the alcohols transformed to aldehydes by an alcohol oxidase (Teal and Tumlinson 1986), and acetates produced by acetyl transferase activity (Jurenka 2004).

The superfamily Tortricidae is a group of leafroller moths distributed throughout the world, and includes many species that are pests of various crops. The brown-headed and green-headed leafrollers of the genera endemic in New Zealand, *Ctenopseustis* and *Planotortrix*, respectively, contain species that are pests of horticultural crops (Wearing et al. 1991). The sex pheromones used by species within these genera have attracted considerable attention due to their use of monounsaturated acetates with double bonds in the *Z* configuration at positions that are unusual compared with pheromones components of other moths, including those within the Tortricidae. These components include tetra- and hexadecenyl acetates with desaturation at the 5, 7, 8, 9, and 10 positions (Clearwater et al. 1991; Foster et al. 1991; Foster and Roelofs 1987; Young et al. 1996), whereas the 11 position commonly is utilized in other tortricid moths (Blomquist et al. 2005; Jurenka 2004).

Within *Ctenopseustis*, *C. obliquana* uses a binary pheromone of (*Z*)-8-tetradecenyl acetate (Z8-14:OAc) and (*Z*)-5-tetradecenyl acetate (Z5-14:OAc) in a 4:1 ratio (Clearwater et al. 1991; Young et al. 1985), whereas *C. herana* uses Z5-14:OAc as its sole pheromone component (Foster and Roelofs 1996). In *C. obliquana*, Z8-14:OAc is biosynthesized from stearic acid (18:Acyl) that is chain shortened to 16:Acyl, followed by Δ10-desaturation to produce the intermediate Z10-16:Acyl moiety, which is further chain shortened to Z8-14:Acyl before reduction and final acetylation (Foster and Roelofs 1988). To date, six desaturases have been identified as expressed within the pheromone gland of *Ctenopseustis* and *Planotortrix* species, including two Δ9-, a Δ10-, a Δ6-desaturase, a terminal desaturase, and a desaturase thought to be non-functional (Albre et al. 2012; Hao et al. 2002). In *C. herana, desat5,* which encodes the Δ10-desaturase, is down-regulated in the pheromone gland, resulting in no Z8-14:OAc present in the sex pheromone (Albre et al. 2012). The sole pheromone component used by *C. herana*, Z5-14:OAc is synthesized directly from tetradecanoic (myristic) acid by Δ5-desaturation, followed by reduction and acetylation (Foster and Roelofs 1996). A similar pathway presumably is responsible for the biosynthesis of Z5-14:OAc in *C. obliquana*, but this has not been investigated, and in neither species has the desaturase gene encoding a Δ5-desaturase been discovered.

In this study, we identified and functionally characterized a novel desaturase gene (*desat7*) expressed in the pheromone glands of female *C. obliquana* and *C. herana*. When the desaturase encoded by this enzyme was heterologously expressed in yeast, it was found to display a unique Δ5-desaturase activity on myristic acid to produce Z5-14:Acyl.

## Methods and Materials

### Insects and Chemicals

*C. obliquana,* and *C. herana* were obtained from Plant & Food Research (previously HortResearch and before that DSIR) insect rearing facility at the Mt Albert Research Centre, Auckland, New Zealand. The history of these strains is reported in Newcomb and Gleeson (1998). Insects were reared on a 16:8 hr l:D cycle, with larvae reared at 20 °C, and pupae and adults at 18 °C. Larvae were reared individually on a general-purpose diet as described in Albre et al. (2012). Tetradecanoic acid (14:COOH) was bought from Larodan Fine Chemicals AB (Limhamn, Sweden) and contained no detectable amounts of ∆5 unsaturated tetradecenoic acid. Methyl esters of (*Z*)-5 and (*E*)-5-tetradecenoate (Z5-14:ME and E5-14:ME) were synthesized from their corresponding alcohols (purchased from Pherobank) as described in Lassance et al. (2010).

### Identification of *desat7*, Phylogenetic Analysis, and Bioinformatics

The procedures for the isolation of RNA and DNA are described in Albre et al. (2012). Briefly, pheromone glands of 2–3 day-old virgin females were dissected on ice and stored at −80 °C. RNA was extracted using a modified trizol method (Life Technologies, Carlsbad, CA, USA) and used as template for the construction of RNAseq libraries using Illumina’s standard protocols, and sequenced at Macrogen (Seoul, South Korea). Resulting sequences were assembled and made available as blast sets for further analysis. Blast searches using a range of insect desaturase sequences then were performed to identify any further desaturases not previously described in Albre et al. (2012). Novel desaturases that were identified were made full length using RACE, amplified in their entirety from antennal cDNA by PCR and resequenced for confirmation.

For phylogenetic analyses, a common lepidopteran Δ11-desaturase (JX679209) from the turnip moth *Agrotis segetum* (unpublished data) was used as a query to identify typical pheromone biosynthetic fatty acyl desaturase homologs using BLASTp from GenBank non-redundant (nr) protein database (NCBI http://www.ncbi.nlm.nih.gov) by different approaches in order to obtain a broad set of desaturase homologues from as many organisms as possible. The initial BLASTp searches were performed using default settings, and 100 sequence hits were downloaded. Searches were conducted with the aforementioned *A. segetum* desaturase and, once identified, with Desat7. A second round of blast searches were conducted this time excluding Lepidoptera and Drosophila protein sequences from the search, and just the 20 best hits were downloaded. The third and fourth sets of searches were performed in the same way as the second, with the former excluding insect sequences from the database, and the latter excluding arthropods. Duplicate hits and synthetic sequences were removed manually. *Ctenopseustis obliquana* and *C. herana* Desat7 were included with the desaturase set and aligned using ClustalW2 (Chenna et al. 2003; Larkin et al. 2007) in MEGA5 (Tamura et al. 2011). Phylogenetic trees were built using JTT distances by the Neighbor-Joining method with gaps treated by pairwise deletion, and trees bootstrapped with 1500 bootstrap replicates. The *C. obliquana* and *C. herana* desaturase sequences were analyzed with the subcellular localization prediction tools Euk-mPLoc 2.0 (Chou and Shen 2010) and ProtComp 9.0 (Softberry, USA).

### Quantitative RT-PCR and Analysis

Pheromone glands and adjacent abdominal tissue were dissected on ice from 2 to 3 day-old virgin females and stored at −80 °C. RNA was extracted from tissue samples from ten individuals at a time to produce each biological replicate. Total RNA isolation and cDNA synthesis were carried out according to the methods described in Albre et al. (2012). The levels of expression of *desat7* were determined using the primers Co-d7-F2 (5′-CCGGCGTTCACCGCTACTGG-3′) and Co-d7-R2 (5′-AAGAAGAAGCCGCGGGTCGC-3′), alongside those of the housekeepers *α-tubulin*, *actin* and *elongation factor 1-α* using primers described in Albre et al. (2012). Each pool of pheromone glands and corresponding body tissues was tested for levels of expression for each gene, with three technical replicates conducted for each of the three biological replicates. Quantitative RT-PCRs (qPCRs) contained 4 μl cDNA, 5 μl of 2× Roche SYBR green Master Mix (Roche, Basil, Switzerland), and 0.5 μM of each primer to a final volume of 10 μl. The PCR cycling conditions were as follows: 2 min at 95 °C, followed by 45 cycles of 15 sec at 95 °C, 30 sec at 60 °C, and 30 sec at 72 °C. A final dissociation curve analysis was added (15 sec at 95 °C, 15 sec at 60 °C, and a gradual heating to 95 °C at 0.01 °C/sec) to determine the purity of the products.

The relative expression of each gene was calculated using a modified version of the ∆Cq method (Pfaffl 2001; Ramakers et al. 2003). Because the efficiency of a given primer set varied between cDNA samples, resulting Cq values for a particular gene and sample were corrected using the formula (E_MAX_) ^Cq^_corrected_ = (E_sample ‘X’_)^Cq^_sample ‘X’_, where E_MAX_ equals the highest efficiency for a primer pair from all samples, E_sample ‘X’_ equals the efficiency of that primer pair in sample ‘X’, Cq_sample ‘X’_ equals the measured Cq value for sample ‘X’, and Cq_corrected_ equals the corrected Cq value for sample ‘X’. A normalization factor was determined for each sample by averaging the corrected Cq values for the three reference genes from that sample. The relative expression of each gene to the normalization factor for each sample was calculated using the formula (E_MAX_)^(∆Cq)^, where E_MAX_ equals the highest efficiency for a particular primer set, and ∆Cq equals the difference between the Cq of the primer set in that sample and the normalization factor for that sample.

### Construction of pYEXCHT-desat7

The gene-specific primers pTortD5fls (5′-CGGGATCCGTCATGGGTTTTGTGACTCCACTTAAATGG-3′) and pTortD5fl2as (5′-GCGAATTCTTAGTGCTCTGAGCCTATTGGTGCAAACTG-3′) were designed manually in BioEdit (Hall 1999) to include the start/stop codon of the *C. obliquana* and *C. herana desat7* ORFs, as well as a BamHI recognition site (sense primer) or an EcoRI recognition site (antisense primer). The full-length *desat7* alleles named 26 and 38 from *C. obliquana* were PCR amplified from 100 ng cDNA or plasmid DNA using Phusion® High-Fidelity PCR Master Mix (New England Biolabs) with GC buffer under the following cycling conditions: 98 °C for 1 min, 98 °C 10 sec–60 °C 10 sec–72 °C 45 sec for 35 cycles, a final extension step at 72 °C for 1 min, and hold at 4 °C. The products were ligated into pYEXCHT (Patel et al. 2003) with the CloneJET™ PCR Cloning Kit according to the instructions provided by the manufacturer (Thermo Scientific, Waltham, MA, USA), and cloned into One Shot® TOP10 Chemically Competent *E. coli* (Life Technologies). The *C. herana* ORF was synthesized via GeneArt® Gene Synthesis (Life Technologies) to include a BamHI site at the upstream region of the start codon and an EcoRI site downstream of the stop codon. All ORFs were restricted from positive clones in a BamHI and EcoRI double digestion as instructed by the manufacturer (Promega). The reaction was analyzed on a 1 % TAE agarose gel by gel electrophoresis, and the bands corresponding to *desat7* were restricted and prepared using the Wizard® SV Gel and PCR Clean-Up System (Promega, Madison, WI, USA). The sequences were ligated into BamHI/EcoRI digested pYEXCHT in a 1:1 vector:insert ratio using T4 DNA Ligase (Thermo Scientific) and cloned into *NEB* 5-alpha Competent *E. coli* as described by the manufacturer (New England Biolabs, Ipswich, MA, USA). Colonies were analyzed by colony PCR using the primers pYEX-s (5′-AATATACCTCTATACTTTAACGTC-3′) and pYEX-as (5′-ACCGAGGAGAGGGTTAGGGAT-3′), as well as pYEX-s and pTortD5fl2as, and those positive for both were sequenced to confirm the presence of the *desat7* insert.

### Functional Assay of *desat7* in *Saccharomyces cerevisiae*

Verified pYEXCHT-*desat7* constructs were transformed into the *S. cerevisiae* double mutant (*MATa elo1::HIS3 ole1::LEU2 ade2 his3 leu2 ura3*) according to the instructions provided in the *S. c.* EasyComp™ Transformation Kit (Life Technologies) and placed under selection on YNB –ura –leu 1 % tergitol 2 % glucose 0.01 % adenine, and 0.5 mM oleic acid agar plates. A single *desat7* yeast transformant colony was inoculated into 4 ml of selective media and grown for 48 h at 30 °C, 300 rpm. The cultures were diluted to OD_600_ of 0.4 in 20 ml of selective media with the addition of 2 mM CuSO_4_, and 0.5 mM myristic acid, or with equal concentrations of fatty acids between C8 to C16 in a total amount of 1 mM, and the cultures grown on for an additional 48 hr. The yeast cells were harvested by centrifugation, and lipids were extracted with 500 μl of chloroform:methanol (2:1, v:v) for 1 hr at room temperature, prior to evaporation of the solvent under a gentle N_2_ stream. The lipids were subjected to base methanolysis to convert all fatty-acyl moieties into the corresponding methyl esters and dissolved in *n*-hexane prior to GC/MS analysis, as described in Liénard et al. (2008). For GC/MS analysis, a Hewlett Packard HP 5890II GC system (Agilent), coupled to an HP 5972 mass selective detector (Agilent) and equipped with a medium-polar INNOWax column (100 % polyethylene glycol, 30 m 60.25 mm I.D., film thickness 0.25 mm, Agilent Technologies) was used. The GC/MS was operated in electron impact mode (70 eV), the injector was configured in splitless mode at 220 °C, and helium was used as carrier gas (velocity: 30 cm/sec). The oven temperature was maintained for 2 min at 50 °C and increased at a rate of 10 °C/min up to 220 °C, and held for 20 min. In order to localize double bond positions in monoenes, dimethyl-disulfide (DMDS) adducts were prepared according to Buser et al. (1983), and the samples were analyzed with a Hewlett Packard HP 6890 GC system coupled to an HP 5973 mass selective detector (Agilent). The GC was equipped with an HP1-MS column (100 % methyl siloxane; 30 m 3 0.25 mm, df: 0.25 μm; Agilent), and helium was used as carrier gas (velocity: 32 cm/sec). The oven temperature was set to 55 °C (or 80 °C) for 2 min, then increased by 10 °C/min up to 250 °C, followed by a hold at 250 °C for 10 min, and then increased by 20 °C/min up to 300 °C followed by a hold at 300 °C for 5 min.

## Results

### Phylogenetic Analysis and Prediction of Subcellular Localization

**Fig. 1:**
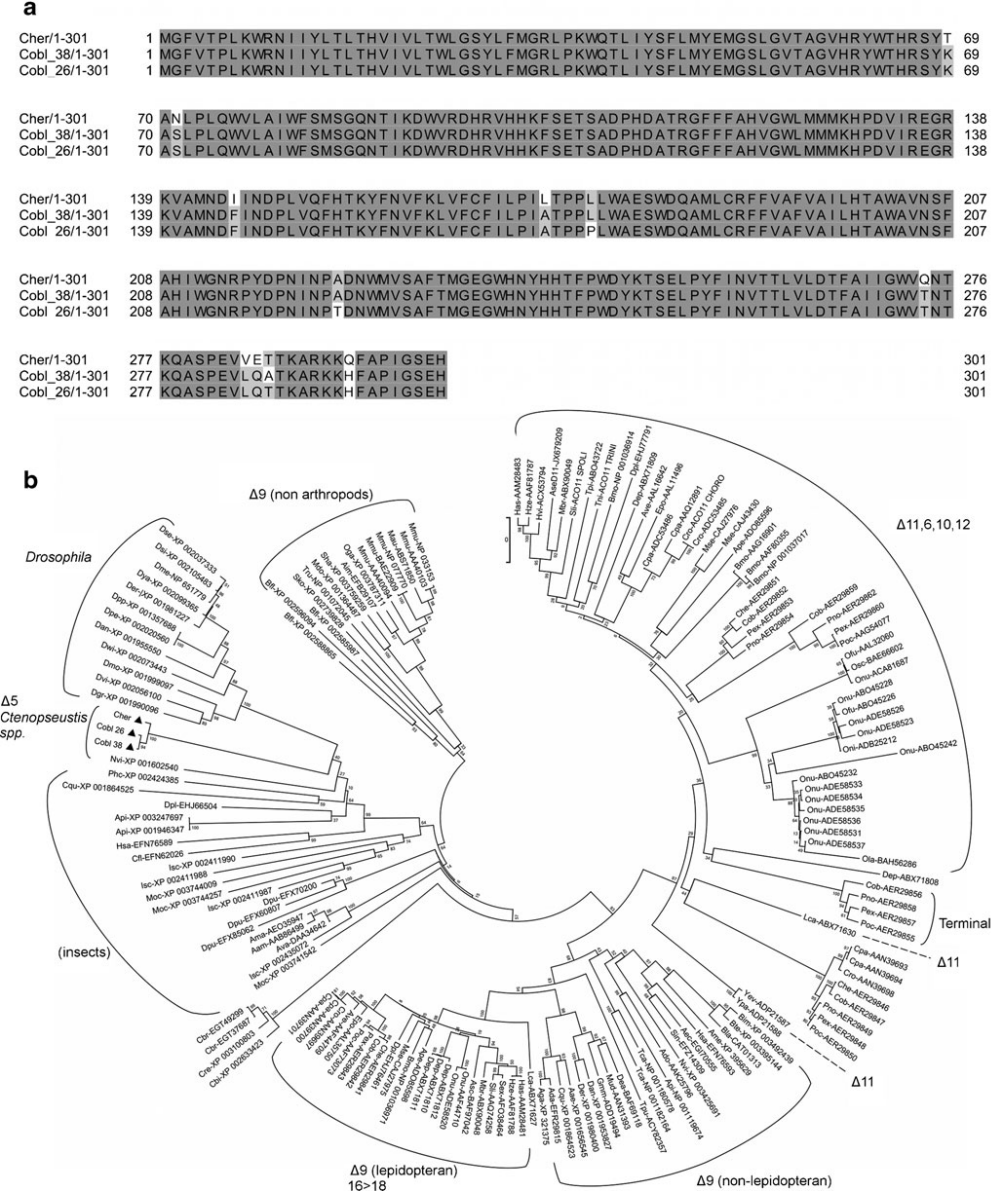
Sequence and phylogenetic analysis of Desat7. **a** An alignment of Desat7 isoforms from *Ctenopseustis obliquana* and *C. herana*. Amino acid differences are displayed in *white or light grey*. **b** A phylogenetic tree including Desat7 (marked with *black triangles*) together with other moth desaturases as well as desaturases from other organisms (SI Table 1). Groups containing desaturases with similar activities are indicated with *brackets* and all bootstrap values are noted at the node of each branch

A new gene predicted to encode a desaturase was identified from the transcriptomes of female pheromone glands from *C. obliquana* and *C. herana* using Blast searches with insect desaturases. Two alleles named 26 and 38 were recovered from *C. obliquana* (GenBank accession numbers KF651145 and KF651144, respectively), and one allele was recovered from *C. herana* (GenBank accession number KF651146). The predicted full-length protein sequences of the new desaturase (Desat7) are 301 amino acids in length in both species, and the proteins are 98 % (*C. obliquana* 26–38) 97 % (*C. obliquana* 38 – *C. herana*), and 96 % (*C. obliquana* 26 – *C. herana*) identical at the amino acid level (Fig. 1a). Blast searches confirmed that Desat7 contains all the features of a desaturase, i.e. Delta9-FADS-like domains, His-boxes, and Membrane-FADS-like domains. Both Euk-mPLoc 2.0 and ProtComp predicted Desat7 to be located in the endoplasmic reticulum, as is predicted for all other desaturases (Shanklin and Cahoon 1998; Stuckey et al. 1990; Tocher et al. 1998).

Desat7 shares 57 % amino acid identity with its most closely related desaturase, a putative fatty acid desaturase from the butterfly *Danaus plexippus* (EHJ66504). The predicted Desat7 protein sequences clustered in a group of their own on a phylogenetic tree of insect desaturases, with the most closely related lineages being a group of putative Δ9- or Δ11-desaturases from *Drosophila* and another group of predicted desaturases from other insects (Fig. 1b). Desat7 did not fall within any other clades previously associated with desaturases involved in pheromone biosynthesis in moths.

### Quantitative RT-PCR of *desat7*

**Fig. 2:**
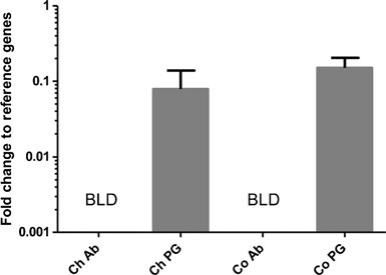
Quantitative RT-PCR of desat7 in pheromone gland and abdominal tissues of *Ctenopseustis obliquana* and *C. herana* adult females. *Co Ctenopseustis obliquana*, *Ch C. herana*, *PG* pheromone gland, *Ab* abdominal tissue, *BLD* below limits of detection. *Error bars* are standard errors of the means of three biological replicates

The expression of *desat7* in adult female pheromone gland and abdominal tissues in *C. obliquana* and *C. herana* was assessed by qPCR. Transcripts of *desat7* were detected in the pheromone glands of females of both *C. obliquana* and *C. herana*, but not in adjacent abdominal tissue where expression levels were below the limits of detection (Fig. 2).

### Heterologous Expression of *desat7* in Yeast

**Fig. 3:**
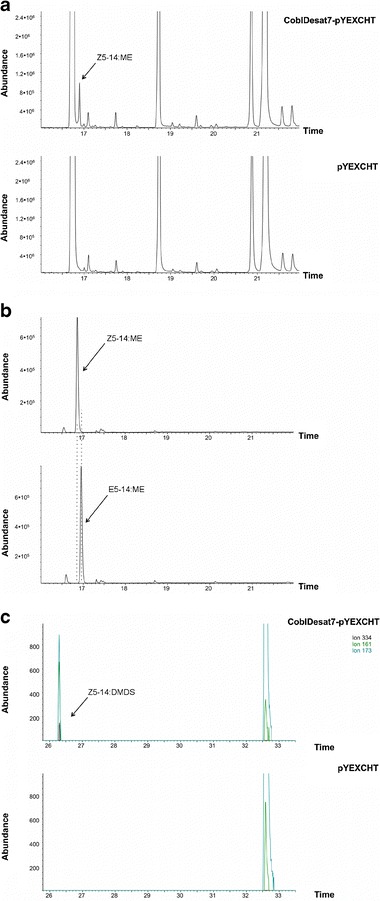
GC/MS Analyses of products from heterologous expression of Desat7 in *Saccharomyces cerevisia.e* **a** The *top panel* corresponds to the base methanolysed extract of yeast expressing Desat7 from *Ctenopseustis obliquana*, allele 38. The enzyme product (*Z*)*-*5-tetradecenoate methyl ester (Z5-14:ME) is indicated. The *bottom panel* shows the extract from the negative control with no Z5-14:ME produced. **b** The reference compounds Z5-14:ME (*top panel*) and (*E*)*-*5-tetradecenyl methyl ester (E5-14:ME) (*bottom panel*). The difference in retention time is indicated with *dashed lines*. **c** DMDS adducts of the extract from **a**, with the extracted ions 334, 173, and 161 indicative of the Δ5 double bond (*top panel*) that is not present in the negative control (*bottom panel*)

The *desat7* orthologues from *C. obliquana* and *C. herana* were successfully transformed into the double mutant strain of *S. cerevisiae*. When the samples from both *C. obliquana* allele 38 and *C. herana* desat7 constructs were extracted and analyzed by GC/MS, a peak appeared with the characteristic ions for an unsaturated C14 methyl ester: 240, 208, and 166 (Fig. 3a). The *C. obliquana* allele 26 failed to produce a corresponding peak. The retention time for the peak corresponded to that for the reference compound Z5-14:ME (Fig. 3b), as determined by overlaying the chromatograms and comparing the retention times of the two isomers (data not shown). The double bond in the 5-position was further confirmed by analysis of the adducts produced upon DMDS derivatization (Fig. 3c) (characteristic ions *m/z* 334, 173, and 161) (Buser et al. 1983). *S. cerevisiae* naturally produce a range of fatty acids between C6 and C18 (Bardi et al. 1999), and these can be accessed directly by the heterologously expressed desaturase in the double mutant yeast strain (Liénard et al. 2008; Wang et al. 2010). These fatty acids were indeed present, but methyl esters of no other monounsaturated acids were found in any of the extracts, except for the derivative of Z9-18:Acyl that was included in the media to support the growth of the *elo1 ole1* deficient yeast. When saturated C8 to C16 fatty acids were added to the yeast, the only desaturated product found in the cell extract was again Z5-14:ME (SI Fig. 1). If Desat7 was able to desaturate any of the other fatty acids, then the characteristic ions for each would have been detected among the DMDS adducts. Since no other unsaturated products were found in the extract, this suggests that the Desat7 enzyme is not able to use any of these other fatty acids as substrate.

## Discussion

Unraveling the biochemical machinery underlying the production of female moth sex pheromones is fundamental to understanding how mate-recognition systems evolve. The two leafroller moth species *C. obliquana* and *C. herana* both produce Z5-14:OAc as, or as a part of, their sex pheromone. Evidence from labeling studies has pointed to the presence of a desaturase with Δ5-desaturase activity on 14:CoA (Foster 1998; Foster and Roelofs 1996), but so far no gene has been linked to this activity or characterized *in vivo*. This led us to revisit the pheromone gland transcriptomes of these species and functionally characterize a new desaturase gene named *desat7*.

The *desat7* gene is expressed in the pheromone glands of females of both *C. obliquana* and *C. herana*. Furthermore, the encoded protein sequence of the newly identified desaturase, Desat7, shows all the conserved features associated with a desaturase, and like other desaturases is predicted to be located in the endoplasmic reticulum. Based on phylogenetic analysis, Desat7 is highly distinct from other characterized moth pheromone biosynthetic desaturases (Fig. 1a–b), with the most closely related lepidopteran pheromone desaturases being 57 % identical at the amino acid level.

When heterologously expressed in *S. cerevisiae,* both the *C. obliquana* allele 38 of Desat7 and the *C. herana* Desat7 were able to catalyze the formation of a Δ5 double bond into 14:Acyl (Fig. 2a–c), yielding the pheromone intermediate Z5-14:ME. This activity is consistent with what was observed by Foster and Roelofs (1996) within the pheromone glands of female *C. herana*. We found no major discernable differences in specificity of the *C. obliquana* and *C. herana* Desat7 desaturases*,* indicating that the differences in the amino acid sequence of these proteins are likely to be functionally neutral. The *C. obliquana* allele 26 of *desat7* may represent a nonfunctional allele or synthetic nonfunctional mutant generated during the PCR. These results demonstrate that it is likely that *desat7* is solely responsible for producing Z5-14:OAc in both of these species.

The only difference between the pheromone biosynthetic pathways in *C. obliquana* and *C. herana* is the expression of *desat5*, which encodes a Δ10-desaturase and is responsible for the production of Z8-14:OAc (Albre et al. 2012). In *C. herana, desat5* is down-regulated so only Z5-14:OAc is produced. In comparison with the *Ctenopseustis* species, the biosynthesis of Z5-14:OAc in the closely related *Planotortrix excessana* involves Δ9-desaturation of stearic acid followed by chain-shortening (Foster 1998). Efforts to locate *desat7* orthologues within available databases of *P. excessana* and *P. octo* have, to date, proved unsuccessful (data not shown).

The Δ5-desaturase activity of Desat7 is unusual among moths and has not been described previously. The enzyme also seems to have a strict specificity for myristic acid as a substrate. The yeast functional assay coupled with DMDS derivatization is a sensitive method for the identification of all monounsaturated fatty acids (Buser et al. 1983), and apart from myristic acid, neither the natural fatty acid substrates occurring in the yeast cells nor supplemented fatty acids from C8–C16 were desaturated by the enzyme.

The desaturases involved in moth pheromone biosynthesis are believed to have evolved from one or many gene duplication events that occurred after the split of the Diptera and Lepidoptera. The novel activities of desaturases in pheromone biosynthesis are due to the evolution of many Lepidoptera-specific desaturase lineages that are distinct from their metabolic desaturase counterparts (Liénard et al. 2008). The six desaturases that have been found previously in these *Ctenopseustis* species all cluster within well-known groups of lepidopteran Δ9- and Δ11-desaturases (Albre et al. 2012; Fig. 1b). It is interesting that Desat7 does not cluster within these well-known moth desaturases, and instead resides in a subgroup of its own alongside other insect desaturase clades (Fig. 1b). This analysis indicates that the gene duplication event that yielded the ancestral *desat7* likely occurred early during the evolution of the moth desaturase lineages. In conclusion, we have uncovered yet another group of desaturases that have evolved a role in biosynthesis of pheromone components in the Lepidoptera.

## Electronic supplementary material

Below is the link to the electronic supplementary material.

SI Table 1 (XLSX 13.7 kb)

SI Fig. 1 *The heterologous expression of Desat7 in* Saccharomyces cerevisiae *including additional fatty acids* a) DMDS adducts prepared from *Ctenopseustis herana* Desat7 yeast cells that have been supplemented with a mixture of the saturated fatty acids ranging from C8 to C16. The peak corresponding to Z5-14:ME is emphasized with an arrow. b) The mass spectrum of the peak corresponding to Z5-14:ME in a). The ions *m/z* 334, 173, and 161 are indicative of the Δ5 double bond, which was the only detected double bond in the extract apart from the supplemented Z9-18:COOH. (PPT 1.02 MB)
