# Supplementary figures and images for: A Novel Fatty Acyl Desaturase from the Pheromone Glands of Ctenopseustis obliquana and C. herana with Specific Z5-Desaturase Activity on Myristic Acid
Source: J Chem Ecol. 2014 Jan 11;40(1):63–70. doi: 10.1007/s10886-013-0373-1 (PMC3909261; doi:10.1007/s10886-013-0373-1)

## Slide 1
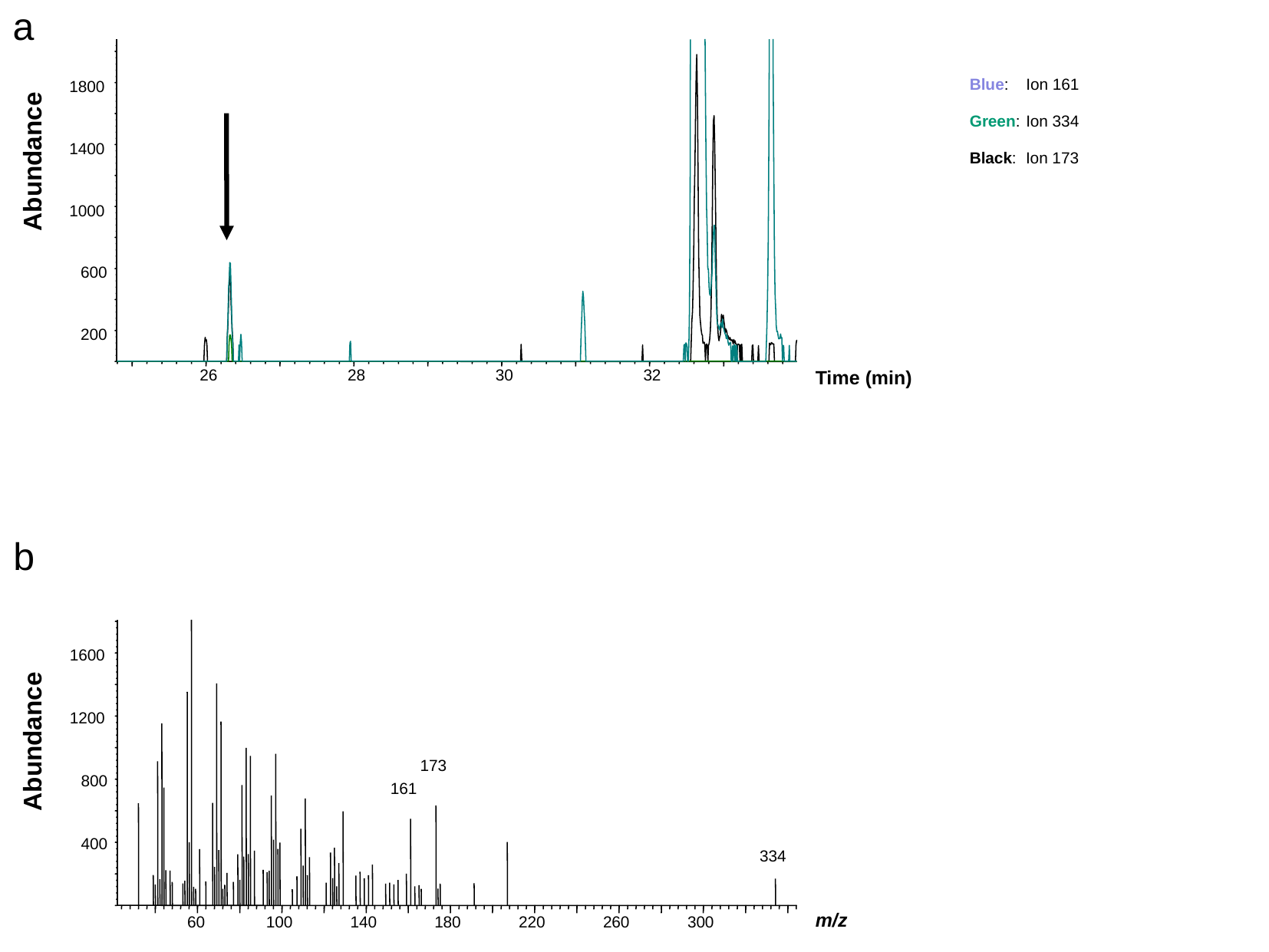

a
Blue: 	Ion 161
1800
Green: 	Ion 334
1400
Abundance
Black: 	Ion 173
1000
600
200
26
28
30
32
Time (min)
b
1600
1200
Abundance
173
800
161
400
334
m/z
60
100
140
180
220
260
300

Supplement: Supplementary file 2 — The heterologous expression of Desat7 in Saccharomyces cerevisiae including additional fatty acids a) DMDS adducts prepared from Ctenopseustis herana Desat7 yeast cells that have been supplemented with a mixture of the saturated fatty acids ranging from C8 to C16. The peak corresponding to Z5-14:ME is emphasized with an arrow. b) The mass spectrum of the peak corresponding to Z5-14:ME in a). The ions m/z 334, 173, and 161 are indicative of the Δ5 double bond, which was the only detected double bond in the extract apart from the supplemented Z9-18:COOH. (PPT 1.02 MB) [file 10886_2013_373_MOESM2_ESM.ppt]
